# Monomeric Ti(IV) homopiperazine complexes and their exploitation for the ring opening polymerisation of *rac*-lactide

**DOI:** 10.1186/1752-153X-7-135

**Published:** 2013-08-06

**Authors:** Stuart L Hancock, Mary F Mahon, Matthew D Jones

**Affiliations:** 1 Department of Chemistry, University of Bath, Claverton Down, Bath BA2 7AY, UK

**Keywords:** Titanium, Polymerisation, Lactide, Catalysis

## Abstract

**Background:**

The area of biodegradable/sustainable polymers is one of increasing importance in the 21st Century due to their positive environmental characteristics. Lewis acidic metal centres are currently one of the most popular choices for the initiator for the polymerisation. Thus, in this paper we report the synthesis and characterisation of a series of monometallic homopiperazine Ti(IV) complexes where we have systematically varied the sterics of the phenol moieties.

**Results:**

When the *ortho* substituent of the ligand is either a Me, *t*Bu or amyl then the β-cis isomer is isolated exclusively in the solid-state. Nevertheless, in solution multiple isomers are clearly observed from analysis of the NMR spectra. However, when the *ortho* substituent is an H-atom then the *trans*-isomer is formed in the solid-state and solely in solution. The complexes have been screened for the polymerisation of *rac*-lactide in solution and under the industrially preferred melt conditions. Narrow molecular weight material (PDI 1.07 – 1.23) is formed under melt conditions with controlled molecular weights.

**Conclusions:**

Six new Ti(IV) complexes are presented which are highly active for the polymerisation. In all cases atactic polymer is prepared with predictable molecular weight control. This shows the potential applicability of Ti(IV) to initiate the polymerisations.

## Background

**Figure 1:**
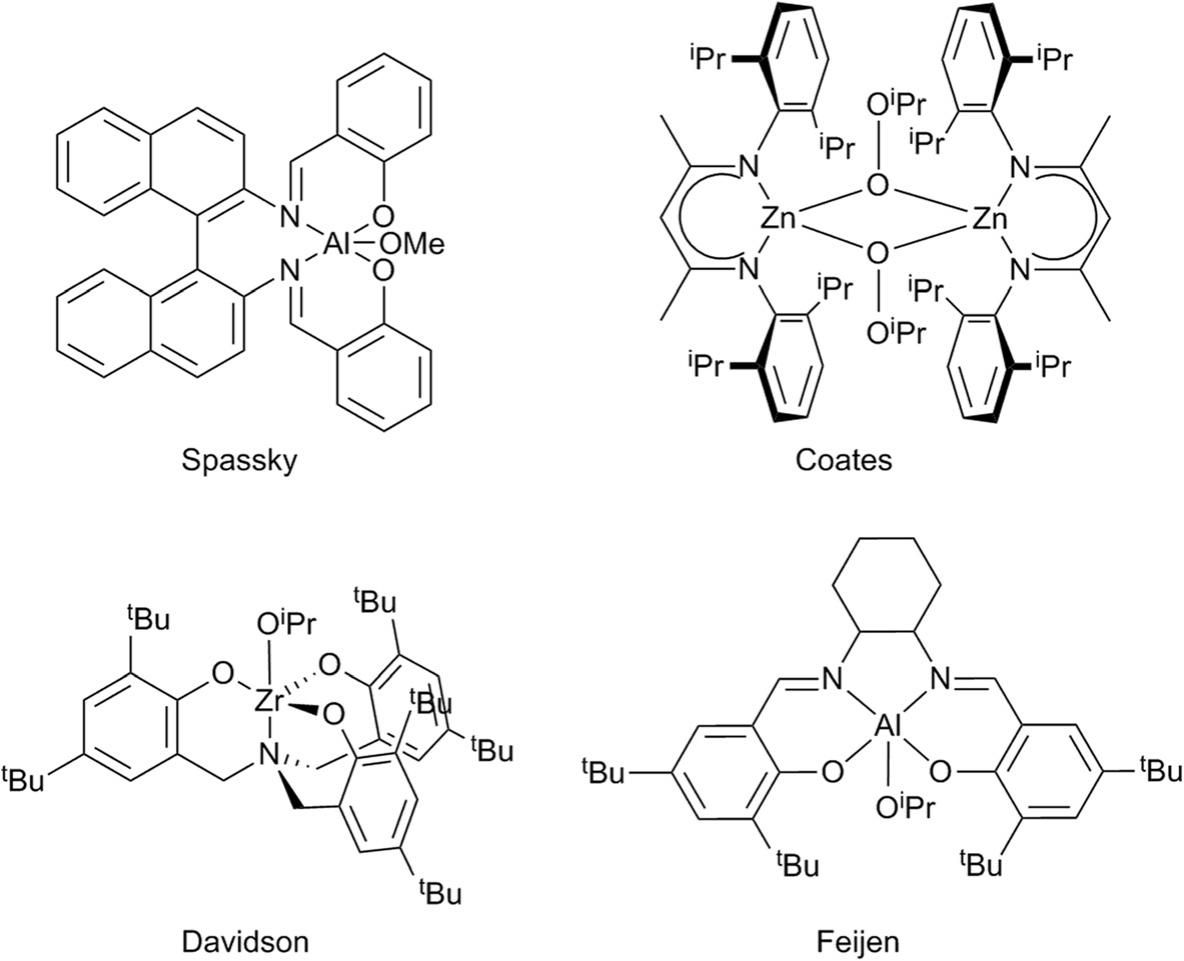
**Examples of initiators for the ROP of *rac*-lactide.**

As part of our on-going studies into the chemistry of group 4 metals and homo/piperazine derived salan ligands [1-3] in this paper we report the synthesis and characterisation of series of monometallic complexes based on the homopiperazine backbone. This ligand family has also been applied to Fe(III) [4,5], Cu(II) [6], Ni(II) [6] and Mo(VI) [7] metal centres. Typically these are either monomeric or dimeric structures in the solid-state. These 7-membered ring ligands are under-represented in the literature compared to their 6-membered brothers or their linear amine bis(phenolate) cousins [8-13]. To re-address this imbalance we have previously reported the formation of Ti_2_(O^i^Pr)_6_L or monometallic Zr/Hf(O^i^Pr)_2_L species (containing homopiperazine salan ligands) where, in the monometallic examples, the O^i^Pr moieties are *trans* to one another [3]. Utilising the piperazine derived salan ligands with Zr(IV) and Hf(IV) starting materials leads to unpredictable reactions with no rationale control over the product formed [3]. These complexes have been shown to be effective initiators for the ring opening polymerisation (ROP) of cyclic esters [2,3]. Moreover, we have prepared Al(III) complexes of homopiperazine salan ligands for co-polymerisations of cyclic esters [1]. The rich and unexplored chemistry of this ligand set motivated us to prepare monometallic Ti(IV) complexes for the controlled ROP of *rac*-lactide. The driving force for this work also lies in the attractive properties of the final polymer polylactide (PLA) itself, such as biodegradability, it is produced from annually renewable resources and the fact that the polymer is also biocompatible [14]. These facets have spear-headed research in this area and metals such as Ca(II) [15,16], Mg(II) [17-20], Zn(II) [21-27], Al(III) [28-36], Bi(III) [37], Ti(IV)/Zr(IV) [38-40] and metal-free systems [41-43] have all proved excellent choices in the literature. The controlled polymerisation of *rac*-lactide can lead to either atactic, heterotactic or isotactic PLA the later possessing a significantly higher melting temperature. There is an exigent desire to prepare and characterise new initiators for the ROP of lactide to enhance the already impressive properties of the material. A selection of complexes for the polymerisation of *rac-*lactide is shown in Figure 1. One of the earliest examples of the ROP of *rac*-lactide was by Spassky and co-workers [33], they produced isotactically enriched PLA with an Aluminium Schiff base complex. Then followed seminal studies on Zn-BDI complexes [17], in solution with a monomer:initiator ratio of 200:1 at 20°C heterotactic PLA *P*_*r*_ = 0.90 was produced. There is a desire to move towards melt polymerisations, in the absence of solvent. One of the first examples of this approach was the work of Feijen [35], who produced highly isotactically enriched PLA from *rac*-lactide at 130°C (monomer:initiator 200:1), however to achieve high conversions 48 hours was required. Davidson has shown that it is possible to produce heterotactically (*P*_*r*_ = 0.90) enriched PLA in the melt with a group 4 amine tris(phenolate) complex (monomer:initiator 300:1), near quantitative conversion was achieved after 10 minutes [44].

## Results and discussion

### Complex preparation

**Scheme 1:**
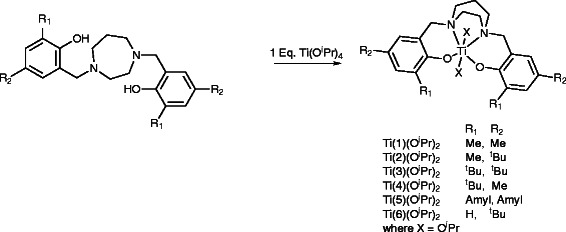
Synthesis of titanium monometallic complexes supported by homopiperazine salan ligands.

Literature preparation methods were utilised to prepare the homopiperazine salan ligands, (**1**-**6**)H_2_[3,45]. The complexes were prepared by a 1:1 reaction of the salan with Ti(O^i^Pr)_4_ at 80°C, this was carried out under a flow of Ar to facilitate the removal of isopropanol to drive the reaction to the formation of the 1:1 complex. The additional heating (80°C) allowed the homopiperazine ring backbone to adopt the thermodynamically unfavourable boat type configuration and furthermore coordinate both phenols and nitrogen centres to a single titanium metal centre, Scheme 1. These complexes were characterised by elemental analysis, ^1^H, ^13^C{^1^H} NMR spectroscopy and where possible single crystal X-ray diffraction.

**Figure 2:**
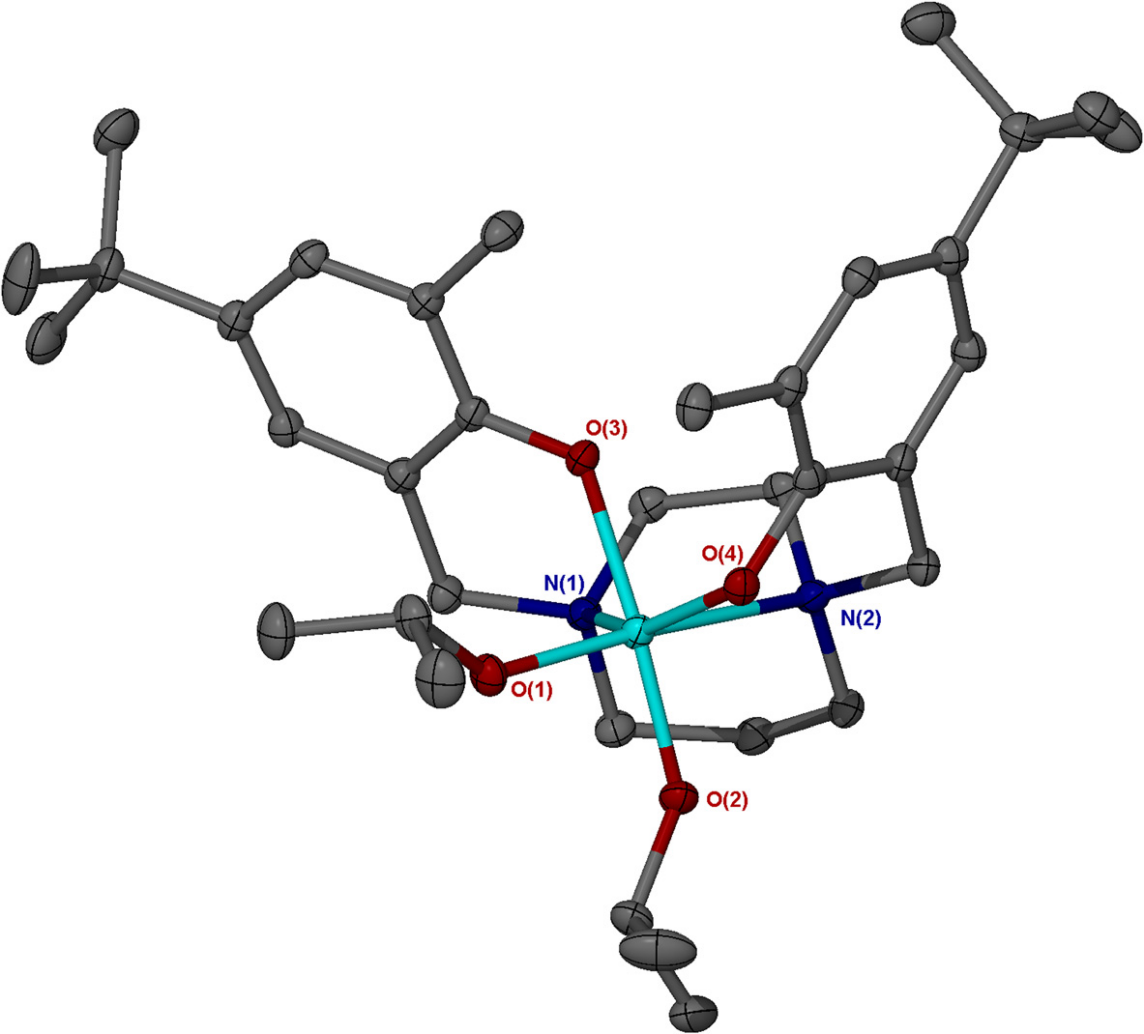
**Solid-state structure for Ti(2)(O**^**i**^**Pr)**_**2 **_**in the β-*cis *configuration.** Ellipsoids are shown at the 30% probability level, hydrogen atoms have been removed for clarity.

**Figure 3:**
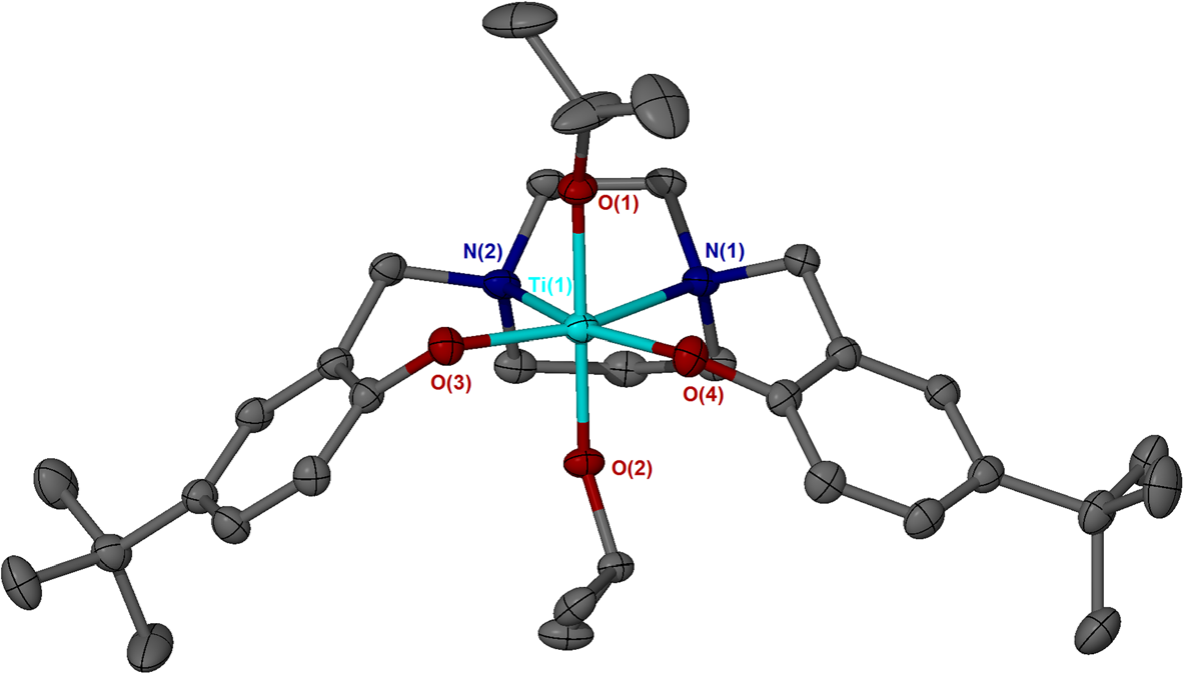
**Solid-state structure for Ti(6)(O**^**i**^**Pr)**_**2 **_**in the *trans*-configuration.** Ellipsoids are shown at the 30% probability level, hydrogen atoms have been removed for clarity.

The solid-state structures Ti(**2**,**4-6**)(O^i^Pr)_2_ have been determined by single crystal X-ray diffraction, and have yielded monometallic complexes with the titanium metal centres adopting a *pseudo* octahedral configuration. The structure obtained for Ti(**2**)(O^i^Pr)_2_ is given as a representative example (Figure 2). Ti(**2,4,5**)(O^i^Pr)_2_ adopt a β-*cis* configuration in the solid-state, this is in contrast to the Zr(IV)/Hf(IV) analogues which formed *trans* complexes. However, with less steric bulk in the *ortho*-phenol position a *pseudo trans*-octahedral titanium complex supported by a homopiperazine salan ligand, Ti(**6**)(O^i^Pr)_2_ (Figure 3), was isolated.

Selected bond lengths (Å) and angles (°) are given in Table 1 for the crystallographically characterised titanium homopiperazine complexes. Those complexes which adopted a β-*cis* configuration {Ti(**2,4-6**)(O^i^Pr)_2_} revealed similar bond lengths and angles. There was no significant difference in the isopropoxide metal (Ti1-O1, Ti1-O2) bond lengths, but phenoxy-metal bond lengths (Ti1-O3, Ti1-O4) were significantly different with the phenoxy *trans* to an isopropoxide exhibiting a longer bond length. The two Ti-N bonds are different with Ti-N *trans* to an isopropoxide being the longer distance. The complexes adopted a distorted octahedral conformation, which is demonstrated by the deviation of the titanium angles from 90° or 180°, for *cis* or *trans* angles respectively. A high degree of variation from the idealistic 90° angle was observed between N1-Ti1-N2, giving angles between 67.90(13) - 68.08(7)°.

**Table 1:** **Selected bond lengths (Å) and angles (°) for Ti(2,4-6)(O**^**i**^**Pr)**_**2**_**, as determined by X-ray diffraction studies**

	**Ti(2)(O**^**i**^**Pr)**_**2**_	**Ti(4)(O**^**i**^**Pr)**_**2**_	**Ti(5)(O**^**i**^**Pr)**_**2**_	**Ti(6)(O**^**i**^**Pr)**_**2**_
**Ti1-O1**	1.8310(17)	1.836(4)	1.812(3)	1.8490(18)
**Ti1-O2**	1.8375(16)	1.833(3)	1.838(3)	1.8323(17)
**Ti1-O3**	1.9568(16)	1.931(3)	1.939(3)	1.9175(19)
**Ti1-O4**	1.8834(17)	1.873(3)	1.892(3)	1.9106(18)
**Ti1-N1**	2.285(2)	2.293(4)	2.298(4)	2.255(2)
**Ti1-N2**	2.349(2)	2.346(4)	2.334(4)	2.268(2)
**N1-Ti1-O1**	101.94(7)	102.78(16)	103.54(15)	86.18(8)
**N1-Ti1-O2**	92.39(7)	89.46(16)	89.09(14)	90.87(8)
**N2-Ti1-O1**	168.57(8)	170.35(16)	171.26(14)	88.51(8)
**N2-Ti1-O2**	84.54(7)	86.07(15)	86.41(14)	87.99(8)
**N1-Ti1-N2**	68.08(7)	67.91(15)	67.90(13)	70.09(8)

The less sterically hindered salan complex with hydrogen atoms at the *ortho* positions adopted a distorted *trans*-octahedral structural configuration {Ti(**6**)(O^i^Pr)_2_} (Figure 3). The two phenoxy-titanium bonds (Ti1-O1, Ti1-O2) are equivalent in length, additionally the two nitrogen-titanium bonds (Ti1-N1, Ti1-N2) are equivalent in length. This is indicative of the structures symmetrical nature. Similar to β-*cis* configurations the *trans*-octahedral structure deviates from an ideal octahedral environment.

**Figure 4:**
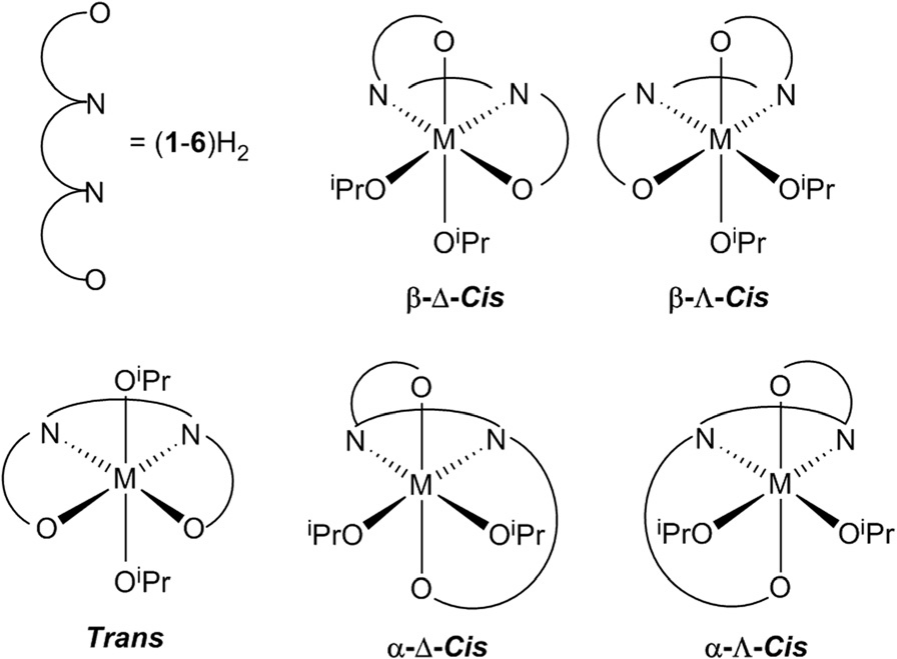
Binding modes of homopiperazine salan ligands.

The solution-state NMR spectra for the monometallic titanium piperazine salan complexes Ti(**1**–**5**)(O^i^Pr)_2_ show that the complexes adopt multiple conformations in solution, unlike the solid-state structures which all showed the β-*cis* conformation. For example for Ti(**1**–**2**)(O^i^Pr)_2_ two conformations are observed in solution. One of the two species in solution is comparatively well defined whereas the other is fluxional. For example isopropoxide –CH_3_ resonances were located at 0.40 ppm and 1.19 ppm (presumably the β-*cis* isomer) and a broad resonance was further observed between 0.45 - 1.55 ppm. The fluxional nature is supported by variable temperature NMR spectroscopy (233 K) where the resonances become much more defined at lower temperatures. These complexes can adopt the α-*cis*, β-*cis*, and *trans* octahedral conformations. Although the Δ and Λ forms of α-*cis* and β-*cis* conformations are possible they are indistinguishable by conventional NMR spectroscopy (Figure 4) [46]. It should be noted that although three octahedral conformations are present the orientation of the homopiperazine ring can further complicate the NMR spectra.

The more sterically hindered complexes Ti(**3**–**5**)(O^i^Pr)_2_, with respect to the *ortho*-phenoxy positions, primarily adopted two conformations. The two conformations can be observed in their NMR spectra. For example for Ti(**3**)(O^i^Pr)_2_ the isopropoxide -CH_3_ region shows doublets at 0.39 ppm, 0.98 ppm and 1.01 ppm which are related to one conformation. The analogous resonances are present from the other conformation at 0.55 ppm, 0.72 ppm, 0.94 ppm, and 0.97 ppm (each a 3H integral) respectively. The two species were present in an approximate 1:0.9 ratio. The same can be observed in the aromatic region where resonances at 6.88 ppm, and 7.25 ppm were attributed to the slightly dominant conformation. The ^1^H NMR resonances are relatively well defined for each conformation at room temperature, it was speculated that the increased steric demands of the ligands reduce fluxionality within the complex when compared to Ti(**1**–**2**)(O^i^Pr)_2_.

The less sterically hindered Ti(**6**)(O^i^Pr)_2_ exclusively formed the *trans* octahedral conformation in solution and the solid-state, as determined by ^1^H/^13^C{^1^H} NMR spectroscopy and single crystal X-ray diffraction. The isopropoxide –CH_3_ protons afforded only two resonances at 0.63 ppm and 1.15 ppm (both 6H integrals) thus consistent with a *trans* octahedral geometry being formed exclusively. This is further supported by the presence of two isopropoxide septets at 3.84 ppm and 4.82 ppm.

### Polymerisation studies

The isolated Ti(**1**–**6**)(O^i^Pr)_2_ complexes were trialled for the ROP of *rac*-lactide in toluene (10 ml) at 80°C at a 100:1 [*rac*-lactide]:[Initiator] ratio (Table 2). Limited activity was observed for this initiator series under these conditions typically achieving low conversions after 24 h. The molecular weights were consistent with one PLA chain per metal; additionally PDI values were low indicating a more controlled polymerisation system than their bimetallic counterparts [2]. The monometallic system is stable at 80°C therefore it was assumed the monometallic species were initiating the polymerisation reaction. Where the initiators were active enough to obtain reliable *P*_*r*_ values a slight isotactic bias was observed.

**Table 2:** **Solution ROP of *rac*-lactide for Ti(1–6)(O**^**i**^**Pr)**_**2 **_**in 10 ml of toluene at 80°C in a 100:1 [ *rac *-lactide]:[initiator]**

	**Time (hours)**	**Conv. (%)**^**a**^	***M***_***n***_^***b***^	**PDI**^**b**^	***P***_***r***_^***c***^
Ti(**1**)(O^i^Pr)_2_	24	23	1250	1.63	-^d^
Ti(**2**)(O^i^Pr)_2_	24	8	-	-	-^d^
Ti(**3**)(O^i^Pr)_2_	24	27	4700	1.17	-^d^
Ti(**4**)(O^i^Pr)_2_	24	12	700	1.01	-^d^
Ti(**5**)(O^i^Pr)_2_	24	50	8200	1.06	0.44
Ti(**6**)(O^i^Pr)_2_	24	32	6950	1.11	0.44

^a^Conversion ascertained by ^1^H NMR spectroscopy. ^b^Molecular weight and PDI determined by GPC (THF) using polystyrene standards. ^c^*P*_*r*_ as calculated from ^1^H NMR homonuclear decoupled spectroscopy in CDCl_3_.^d^*P*_*r*_ could not be accurately determined, strong tacticity was not observed.

Ti(**1**–**6**)(O^i^Pr)_2_ titanium salan complexes were trialled for the ROP of *rac*-lactide without solvent at 130°C at a 300:1 [*rac*-lactide]:[Initiator] ratio (Table 3). Under solvent free conditions these initiators typically achieved 41–60% conversion after 24 h. Despite the presence of two potentially initiating isopropoxide groups per metal the PDI values remained low (PDI < 1.25) at the elevated temperature. The defined structure permits the formation of controlled PLA chains but the lack of flexibility within the molecules causes the initiators to be hindered thus leading to reduced activity. Under melt conditions Ti(**1**–**6**)(O^i^Pr)_2_ complexes produced PLA with a slight heterotactic bias (*P*_*r*_ = 0.51 - 0.63). The steric effects do not seem to significantly alter the polymerisation, with the more bulky amyl substituted complex being more active than the sterically unhindered Ti(**6**)(O^i^Pr)_2_ complex.

**Table 3:** **Solvent free ROP of *rac*-lactide for Ti(1–6)(O**^**i**^**Pr)**_**2 **_**at 130°C in a 300:1 [ *rac *-lactide]:[initiator]**

	**Time (hours)**	**Conv. (%)**^**a**^	***M***_***n***_^***b***^	**PDI**^**b**^	***P***_***r***_^***c***^
Ti(**1**)(O^i^Pr)_2_	24	54	12050	1.07	0.56
Ti(**2**)(O^i^Pr)_2_	24	50	7900	1.19	0.63
Ti(**3**)(O^i^Pr)_2_	24	42	7050	1.14	0.53
Ti(**4**)(O^i^Pr)_2_	24	41	6750	1.10	0.63
Ti(**5**)(O^i^Pr)_2_	24	60	10900	1.14	0.61
Ti(**6**)(O^i^Pr)_2_	24	51	6850	1.23	0.55

^a^Conversion ascertained by ^1^H NMR spectroscopy. ^b^Molecular weight and PDI determined by GPC (THF) using polystyrene standards. ^c^*P*_*r*_ as calculated from ^1^H NMR homonuclear decoupled spectroscopy in CDCl_3_.

## Conclusions

In conclusion a series of six new Ti(IV) complexes have been prepared based on a homopiperazine salan derived ligand. In solution a multitude of species are formed. However, in the solid-state the β-*cis* and *trans* forms were observed, depending on the steric requirement of the ligand. All complexes were active for the ROP of *rac*-LA in solution and under the industrially preferred melt conditions.

### Experimental

Ti(**1**)(O^i^Pr)_2_. **1**H_2_ (0.37 g, 1.00 mmol) and Ti(O^i^Pr)_4_ (0.30 ml, 1.01 mmol) were dissolved in toluene (30 ml) then heated (80°C) and stirred (16 h). The solvent was removed *in-vacuo* and recrystallised from hexane to yield pale yellow crystals (0.14 g, 0.26 mmol, 26%). 2 species identified in the solution state NMR spectra. ^1^H NMR (CDCl_3_): δ 0.40 (3H, d, J = 5.5 Hz, CH_3_), 1.14 (6H, br, CH_3_), 1.19 (3H, d, J = 5.5 Hz, CH_3_), 1.68 (1H, m, CH_2_), 1.88 (1H, m, CH_2_), 2.21 (9H, s, CH_3_), 2.29 (3H, s, CH_3_), 2.42 (2H, br, CH_2_), 2.79 (1H, d, J = 6.0 Hz, CH_2_), 3.11 (1H, d, J = 11.5 Hz, CH_2_), 3.31 (2H, s, CH_2_), 3.60 (1H, d, J = 6.5 Hz, CH_2_), 3.72 (1H, m, CH_2_), 3.95 (1H, m, CH_2_), 4.20 (2H, d, J = 11.0 Hz, CH_2_), 4.46 (1H, m, CH_2_), 4.85 (1H, m, CH_2_), 4.93 (1H, m, CH_2_), 6.68 (2H, s, ArH), 6.91 (1H, s, ArH). 2nd species ^1^H NMR (CDCl_3_): δ 0.45 – 1.45 (12H, br, CH_3_), 2.00 – 2.50 (12H, br, CH_3_), 2.00 – 2.50 (4H, br, CH_2_), 3.00 – 5.00 (10H, br, CH_2_), 3.00 – 5.00 (2H, br, CH), 6.58 (2H, s, ArH ), 6.87 (2H, s, ArH). ^13^C{^1^H} NMR (CDCl_3_): δ 16.5 (CH_3_), 16.9 (CH_3_), 20.8 (CH_3_), 23.0 (CH_2_), 23.7 (CH_2_), 25.9 (CH_3_), 26.1 (CH_3_), 26.3 (CH_3_), 50.8 (br, CH_2_), 55.6 (CH_2_), 58.0 (CH_2_), 59.2 (br, CH_2_), 62.7 (br, CH_2_), 64.1 (CH_2_), 72.1 (CH), 73.2 (CH), 75.7 (CH), 75.9 (CH), 123.4 (ArH), 124.6 (ArH), 125.4 (ArH), 127.4 (Ar), 122.0 – 132.0 (Ar), 131.5 (Ar), 163.0 (ArO). Calc. (%) for C_29_H_44_N_2_O_4_Ti: C 65.41, H 8.33, N 5.26. Found (%), C 65.29, H 8.27, N 5.37.

Ti(**2**)(O^i^Pr)_2_. **2**H_2_ (0.46 g, 1.02 mmol) and Ti(O^i^Pr)_4_ (0.30 ml, 1.01 mmol) were dissolved in toluene (30 ml) then heated (80°C) and stirred (16 h). The solvent was removed *in-vacuo* and recrystallised from hexane to yield pale yellow crystals (0.48 g, 0.78 mmol, 77%). 2 species identified in the solution state NMR spectra. ^1^H NMR (CDCl_3_): δ 0.32 (3H, d, J = 6.0 Hz, CH_3_), 0.87 (6H, br, CH_3_), 1.65 (9H, d, J = 6.0 Hz, CH_3_), 1.26 (36H, s, CH_3_), 1.71 (1H, m, CH_2_), 1.90 (2H, m, CH_2_), 2.16 (4H, br, CH_2_), 2.28 (12H, s, CH_3_), 2.24 (2H, br, CH_2_), 2.80 (1H, d, J = 6.5 Hz, CH_2_), 3.16 (1H, d, J = 11.5 Hz, CH_2_), 3.30 (4H, br, CH_2_), 3.61 (1H, d, J = 6.5 Hz, CH_2_), 3.71 (2H, m, CH_2_), 3.97 (1H, m, CH), 4.20 (2H, d, J = 11.5 Hz, CH_2_), 4.22 (1H, br, CH_2_), 4.45 (1H, m, CH), 4.80 (1H, m, CH), 4.92 (1H, m, CH), 6.74 (2H, br, ArH), 6.86 (2H, s, ArH), 7.05 (2H, br, ArH), 7.11 (2H, s, ArH). ^13^C{^1^H} NMR (CDCl_3_): δ 16.5 (CH_3_), 16.9 (CH_3_), 20.7 (CH_3_), 23.0 (C), 23.7 (C), 25.8 (CH_3_), 26.1 (CH_3_), 26.3 (CH_3_), 55.6 (CH_2_), 58.0 (CH_2_), 59.2 (br, CH_2_), 62.7 (br, CH_2_), 64.1 (CH_2_), 72.1 (CH), 73.1 (CH), 75.7 (CH), 75.9 (CH), 122.9 (Ar), 123.4 (ArH), 124.0 (Ar), 126.7 (br, Ar), 127.9 (ArH), 139.1 (Ar), 163.0 (ArO). Calc. (%) for C_35_H_56_N_2_O_4_Ti: C 68.17, H 9.15, N 4.54. Found (%), C 68.29, H 9.28, N 4.57.

Ti(**3**)(O^i^Pr)_2_. **3**H_2_ (0.54 g, 1.01 mmol) and Ti(O^i^Pr)_4_ (0.30 ml, 1.01 mmol) were dissolved in toluene (30 ml) then heated (80°C) and stirred (16 h). The solvent was removed *in-vacuo* and recrystallised from hexane to yield pale yellow crystals (0.24 g, 0.34 mmol, 34%). 2 species identified in the solution state NMR spectra in an approximate 50:50 ratio, a third species is present in a negligible ratio. ^1^H NMR (CDCl_3_): δ 0.39 (3H, d, J = 6.0 Hz, CH_3_), 0.55 (3H, d, J = 6.0 Hz, CH_3_), 0.72 (3H, d, J = 6.0 Hz, CH_3_), 0.94 (3H, d, J = 6.0 Hz, CH_3_), 0.97 (3H, d, J = 6.0 Hz, CH_3_), 0.98 (3H, d, J = 6.0 Hz, CH_3_), 1.01 (6H, d, J = 6.0 Hz, CH_3_), 1.26 (18H, s, ^t^Bu), 1.28 (18H, s, ^t^Bu), 1.46 (9H, s, ^t^Bu), 1.47 (9H, s, ^t^Bu), 1.48 (18H, s, ^t^Bu), 1.82 (2H, m, CH_2_), 2.23 (3H, m, CH_2_), 2.38 (3H, m, CH_2_), 2.45 (1H, m, CH_2_), 2.72 (2H, m, CH_2_), 3.05 (1H, d, J = 11.5 Hz, CH_2_), 3.23 (2H, d, J = 11.5 Hz, CH_2_), 3.44 (1H, d, J = 14.5 Hz, CH_2_), 3.55 (2H, m, CH_2_), 3.61 (2H, d, J = 6.5 Hz, CH_2_), 3.88 (2H, m, CH_2_), 3.97 (1H, br, CH_2_), 4.01 (2H, d, J = 11.5 Hz, CH_2_), 4.13 (1H, d, J = 11.5 Hz, CH_2_), 4.17 (1H, m, CH_2_), 4.23 (1H, m, CH), 4.28 (1H, m, CH), 4.51 (1H, d, J = 11.5 Hz, CH_2_), 4.55 (2H, m, CH), 6.74 (1H, d, J = 2.0 Hz, ArH), 6.88 (2H, d, J = 2.5 Hz, ArH), 6.90 (1H, d, J = 2.5 Hz, ArH), 7.16 (1H, d, J = 2.5 Hz, ArH), 7.25 (2H, d, J = 2.5 Hz, ArH), 7.27 (1H, br, ArH). ^13^C{^1^H} NMR (CDCl_3_): δ 22.9 (CH_2_), 23.3 (CH_2_), 25.4 (CH_3_), 25.5 (CH_3_), 25.9 (CH_3_), 26.1 (CH_3_), 26.2 (CH_3_), 26.7 (CH_3_), 30.0 (CH_3_), 30.3 (CH_3_), 30.6 (CH_3_), 32.0 (CH_3_), 34.1 (C), 34.2 (C), 35.1 (C), 35.3 (C), 35.5 (C), 35.6 (C), 51.3 (CH_2_), 52.6 (CH_2_), 55.2 (CH_2_), 55.3 (CH_2_), 55.6 (CH_2_), 57.7 (CH_2_), 58.4 (CH_2_), 59.0 (CH_2_), 63.2 (CH_2_), 64.4 (CH_2_), 64.6 (CH_2_), 72.7 (CH), 72.9 (CH), 74.9 (CH), 76.0 (CH), 121.5 (ArH), 122.2 (Ar), 122.9 (Ar), 123.5 (Ar), 123.7 (ArH), 123.8 (ArH), 123.9 (ArH), 123.9 (ArH), 124.0 (ArH), 124.4 (ArH), 124.6 (Ar), 134.1 (Ar), 134.3 (Ar), 136.7 (Ar), 136.8 (Ar), 138.3 (Ar), 138.5 (Ar), 139.4 (Ar), 139.5 (Ar), 159.1 (ArO), 160.0 (ArO), 163.5 (ArO), 164.3 (ArO). Calc. (%) for C_41_H_68_N_2_O_4_Ti: C 70.26, H 9.78, N 4.00. Found (%), C 70.19, H 9.69, N 4.12.

Ti(**4**)(O^i^Pr)_2_. **4**H_2_ (0.46 g, 1.02 mmol) and Ti(O^i^Pr)_4_ (0.30 ml, 1.01 mmol) were dissolved in toluene (30 ml) then heated (80°C) and stirred (16 h). The solvent was removed *in-vacuo* and recrystallised from hexane to yield pale yellow crystals (0.16 g, 0.26 mmol, 26%). 2 species identified in the solution state NMR spectra in an approximate 50:50 ratio. ^1^H NMR (CDCl_3_): δ 0.41 (3H, br, CH_3_), 0.55 (3H, d, J = 6.0 Hz, CH_3_), 0.72 (3H, d, J = 6.0 Hz, CH_3_), 0.98 (3H, d, J = 6.0 Hz, CH_3_), 1.02 (3H, d, J = 6.0 Hz, CH_3_), 1.00 (3H, s, CH_3_), 0.98 (6H, br, CH_3_), 1.43 (54H, s, ^t^Bu), 1.81 (3H, m, CH_2_), 2.15 – 2.30 (7H, br, CH_2_), 2.22 (6H, s, CH_3_), 2.24 (6H, s, CH_3_), 2.25 (6H, s, CH_3_), 2.30-2.45 (6H, m, CH_2_), 2.54 (1H, m, CH_2_), 2.66 (2H, m, CH_2_), 2.78 (1H, br, CH_2_), 3.05 (2H, d, J = 12.0 Hz, CH_2_), 3.22 (2H, d, J = 11.5 Hz, CH_2_), 3.41 (2H, t, J = 13.0 Hz, CH_2_), 3.43 (2H, br, CH_2_), 3.54 (2H, br, CH_2_), 3.68 (2H, d, J = 3.5 Hz, CH_2_), 3.82 (2H, br, CH), 3.98 (3H, br, CH_2_), 4.05 (2H, d, J = 12.0 Hz, CH_2_), 4.12 (2H, m, CH_2_), 4.24 (2H, m, CH), 4.51 (2H, d, J = 14.0 Hz, CH_2_), 4.61 (2H, m, CH), 5.31 (1H, d, J = 13.0 Hz, CH_2_), 6.61 (1H, s, ArH), 6.75 (2H, s, ArH), 6.77 (2H, s, ArH ), 6.81 (1H, s, ArH), 6.97 (1H, s, ArH), 7.01 (4H, s, ArH), 7.09 (1H, s, ArH). ^13^C{^1^H} NMR (CDCl_3_): δ 20.9 (CH_3_), 21.0 (CH_3_), 21.1 (CH_3_), 22.9 (CH_3_), 23.4 (CH_3_), 25.8 (CH_3_), 25.9 (CH_3_), 26.0 (CH_3_), 26.3 (CH_3_), 26.4 (CH_3_), 26.9 (CH_3_), 29.9 (CH_3_), 30.2 (CH_3_), 30.6 (CH_3_), 34.8 (C), 35.0 (C), 35.2 (C), 35.3 (C), 50.9 (CH_2_), 52.6 (CH_2_), 55.2 (CH_2_), 55.5 (CH_2_), 55.7 (CH_2_), 57.8 (CH_2_), 58.4 (CH_2_), 59.1 (CH_2_), 62.9 (CH_2_), 64.3 (CH_2_), 64.4 (CH_2_), 64.7 (CH_2_), 72.7 (CH), 73.0 (CH), 75.1 (CH), 75.9 (CH), 121.8 (Ar), 122.9 (Ar), 124.2 (Ar), 124.6 (Ar), 124.9 (Ar), 125.3 (Ar), 125.7 (ArH), 125.9 (Ar), 126.0 (Ar), 126.9 (ArH), 127.4 (ArH), 127.7 (ArH), 127.8 (ArH), 128.0 (ArH), 128.2 (ArH), 134.8 (Ar), 135.0 (Ar), 137.5 (Ar), 137.6 (Ar), 134.8 (Ar), 159.1 (ArO), 159.9 (ArO), 163.5 (ArO), 164.3 (ArO). Calc. (%) for C_35_H_56_N_2_O_4_Ti: C 68.17, H 9.15, N 4.54. Found (%), C 66.45, H 8.85, N 4.58.

Ti(**5**)(O^i^Pr)_2_. **5**H_2_ (0.60 g, 1.01 mmol) and Ti(O^i^Pr)_4_ (0.30 ml, 1.01 mmol) were dissolved in toluene (30 ml) then heated (80°C) and stirred (16 h). The solvent was removed *in-vacuo* and recrystallised from hexane to yield pale yellow crystals (0.13 g, 0.17 mmol, 17%). 2 species identified in the solution state NMR spectra in an approximate 50:50 ratio. ^1^H NMR (CDCl_3_): δ 0.33 (3H, d, J = 6.0 Hz, CH_3_), 0.55 (3H, d, J = 6.0 Hz, CH_3_), 0.67 (15H, m, CH_3_), 0.76 (12H, m, CH_3_), 0.99 (3H, d, J = 6.0 Hz, CH_3_), 1.01 (3H, d, J = 6.0 Hz, CH_3_), 1.12 (6H, d, J = 6.0 Hz, CH_3_), 1.23 (6H, s, CH_3_), 1.26 (12H, s, CH_3_), 1.29 (6H, s, CH_3_), 1.39 (3H, s, CH_3_), 1.41 (3H, s, CH_3_), 1.45 (9H, s, CH_3_), 1.47 (9H, s, CH_3_), 1.60 (8H, m, CH_2_), 1.60 (8H, m, CH_2_), 1.75 – 1.95 (6H, m, CH_2_), 2.07 (3H, m, CH_2_), 2.22 (4H, m, CH_2_), 2.30 – 2.50 (4H, m, CH_2_), 2.66 (1H, m, CH_2_), 2.78 (2H, m, CH_2_), 3.06 (1H, d, J = 11.5 Hz, CH_2_), 3.20 (2H, d, J = 11.5 Hz, CH_2_), 3.44 (1H, d, J = 14.5 Hz, CH_2_), 3.53 (2H, d, J = 7.5 Hz, CH_2_), 3.62 (2H, d, J = 6.5 Hz, CH_2_), 3.90 (2H, m, CH_2_), 4.00 (1H, br, CH_2_), 4.10 (3H, d, J = 11.5 Hz, CH_2_), 4.22 (1H, m, CH_2_), 4.25 (1H, m, CH), 4.34 (1H, m, CH), 4.54 (1H, d, J = 13.5 Hz, CH_2_), 4.59 (1H, m, CH), 4.68 (1H, m, CH), 6.68 (1H, d, J = 2.0 Hz, ArH), 6.83 (2H, d, J = 2.5 Hz, ArH), 6.85 (1H, d, J = 2.0 Hz, ArH), 7.05 (1H, d, J = 2.0 Hz, ArH), 7. 14 (2H, d, J = 2.5 Hz, ArH), 7.16 (1H, br, ArH). ^13^C{^1^H} NMR (CDCl_3_): δ 9.3 (CH_3_), 9.4 (CH_3_), 9.7 (CH_3_), 9.9 (CH_3_), 22.9 (CH_2_), 23.4 (CH_2_), 25.7 (CH_3_), 25.8 (CH_3_), 26.2 (CH_3_), 26.3 (CH_3_), 26.4 (CH_3_), 27.0 (CH_3_), 27.4 (CH_3_), 27.8 (CH_3_), 27.8 (CH_3_), 28.0 (CH_3_), 28.1 (CH_3_), 28.2 (CH_3_), 28.3 (CH_3_), 28.7 (CH_3_), 28.7 (CH_3_), 28.9 (CH_3_), 29.0 (CH_3_), 29.1 (CH_3_), 29.2 (CH_3_), 29.3 (CH_3_), 32.9 (C), 33.3 (C), 33.8 (C), 37.2 (CH_2_), 37.2 (CH_2_), 37.3 (CH_2_), 37.4 (CH_2_), 37.6 (C), 38.3 (C), 38.5 (C), 38.9 (C), 38.9 (C), 51.1 (CH_2_), 52.5 (CH_2_), 55.4 (CH_2_), 55.6 (CH_2_), 57.6 (CH_2_), 58.2 (CH_2_), 59.0 (CH_2_), 63.2 (CH_2_), 64.7 (CH_2_), 64.8 (CH_2_), 65.2 (CH_2_), 72.7 (CH), 73.0 (CH), 74.8 (CH), 75.9 (CH), 120.9 (Ar), 121.9 (Ar), 123.3 (Ar), 123.4 (ArH), 123.8 (ArH), 124.5 (ArH), 124.6 (ArH), 125.3 (ArH), 125.9 (ArH), 126.5 (ArH), 132.9 (Ar), 133.0 (Ar), 134.9 (Ar), 135.1 (Ar), 136.1 (Ar), 136.2 (Ar), 137.2 (Ar), 137.4 (Ar), 158.8 (ArO), 159.7 (ArO), 163.5 (ArO), 164.1 (ArO). CHN Calc. (%) for C_45_H_76_N_2_O_4_Ti: C 71.40, H 10.12, N 3.70. Found (%), C 71.38, H 9.97, N 3.78.

Ti(**6**)(O^i^Pr)_2_. **6**H_2_ (0.43 g, 1.01 mmol) and Ti(O^i^Pr)_4_ (0.30 ml, 1.01 mmol) were dissolved in toluene (30 ml) then heated (80°C) and stirred (16 h). The solvent was removed *in-vacuo* and recrystallised from hexane to yield pale yellow crystals (0.39 g, 0.66 mmol, 65%). ^1^H NMR (CDCl_3_): δ 0.63 (6H, d, J = 6.0 Hz, CH_3_), 1.61 (6H, d, J = 6.0 Hz, CH_3_), 1.28 (18H, s, ^t^Bu), 1.72 (1H, m, CH_2_), 1.72 (1H, m, CH_2_), 2.19 (1H, m, CH_2_), 2.28 (2H, m, CH_2_), 2.81 (2H, d, J = 6.5 Hz, CH_2_), 3.20 (2H, d, J = 11.5 Hz, CH_2_), 3.61 (2H, d, J = 6.0 Hz, CH_2_), 3.61 (2H, br, CH_2_), 3.84 (1H, m, CH), 4.22 (2H, d, J = 11.5 Hz, CH_2_), 4.82 (1H, m, CH), 6.79 (1H, s, ArH), 6.82 (1H, s, ArH), 7.02 (2H, d, J = 2.0 Hz, ArH), 7.18 (1H, s, ArH), 7.27 (1H, s, ArH). ^13^C{^1^H} NMR (CDCl_3_): δ 23.0 (CH_2_), 26.1 (CH_3_), 26.2 (CH_3_), 31.9 (CH_3_), 34.0 (C), 55.3 (CH_2_), 58.0 (CH_2_), 64.1 (CH_2_), 72.0 (CH), 73.0 (CH), 116.6 (ArH), 123.6 (Ar), 125.9 (ArH), 126.5 (ArH), 139.7 (Ar), 164.1 (ArO). CHN Calc. (%) for C_33_H_52_N_2_O_4_Ti: C 67.33, H 8.90, N 4.76. Found (%), C 67.42, H 8.89, N 4.70.

## Methods

For the preparation and characterisation of metal complexes, all reactions and manipulations were performed under an inert atmosphere of argon using standard Schlenk or glovebox techniques. *rac*-lactide (Aldrich) was recrystallised from toluene and sublimed twice prior to use. All other chemicals were purchased from Aldrich. All solvents used in the preparation of metal complexes and polymerisation reactions were dry and obtained *via* SPS (solvent purification system). ^1^H and ^13^C{^1^H} NMR spectra were recorded on a Bruker 250, 300 or 400 MHz instrument and referenced to residual solvent peaks. Coupling constants are given in Hertz. Elemental analyses were performed by Mr Stephen Boyer, London Metropolitan University. The ligands were prepared according to standard literature procedures [3,45] and the purity confirmed *via*^1^H/^13^C{^1^H} NMR spectroscopy and HR-MS prior to use.

### Polymerisation

For solvent-free polymerisations the monomer:initiator ratio employed was 300:1 at a temperature of 130°C, in all cases 1.0 g of *rac*-lactide was used. After the reaction time methanol (20 ml) was added to quench the reaction and the resulting solid was dissolved in dichloromethane. The solvents were removed *in-vacuo* and the resulting solid washed with methanol (3 × 50 ml) to remove any unreacted monomer. For solution polymerisations a monomer:initiator ratio of 100:1 was used. In all cases 1.0 g of lactide and the appropriate amount of initiator were dissolved in toluene (10 ml) these were placed in a pre-heated oil bath and heated for the desired amount of time. For the melt polymerisation 1.0 g of lactide was used in the absence of solvent. The reaction was quenched by the addition of methanol (20 ml). ^1^H NMR spectroscopy (CDCl_3_) and GPC (THF) were used to determine tacticity and molecular weights (*M*_*n*_ and *M*_w_) of the polymers produced; *P*_*r/m*_ (the probability of heterotactic/isotactic linkages) were determined by analysis of the methine region of the homonuclear decoupled ^1^H NMR spectra [17]. Gel Permeation Chromatography (GPC) analyses were performed on a Polymer Laboratories PL-GPC 50 integrated system using a PLgel 5 μm MIXED-D 300 × 7.5 mm column at 35°C, THF solvent (flow rate 1.0 ml/min). The polydispersity index (PDI) was determined from *M*_*w*_/*M*_*n*_ where *M*_n_ is the number average molecular weight and *M*_*w*_ the weight average molecular weight. The polymers were referenced to polystyrene standards.

### Single crystal diffraction

All data were collected on a Nonius kappa CCD diffractometer with MoKα radiation, λ = 0.71073 Å, see Table 4. T = 150(2) K throughout and all structures were solved by direct methods and refined on *F*^*2*^ data using the SHELXL-97 suite of programs [47]. The data as cif format are given in supporting information as Additional file 1. Hydrogen atoms, were included in idealised positions and refined using the riding model. Refinements were generally straightforward with the following exceptions and points of note. Ti(**4**)(O^i^Pr)_2_ despite copious recrystallisation efforts the *R*_*int*_ was higher than desirable. Ti(**5**)(O^i^Pr)_2_ one isopropoxide is disordered over two positions in a 60:40 ratio and despite copious recrystallisation efforts the *R*_*int*_ was higher than desirable. Ti(**6**)(O^i^Pr)_2_ one isopropoxide is disordered over two positions in a 60:40 ratio, the CH_3_ groups of one *t*Bu are disordered over two positions in a 60:40 ratio and one toluene is disordered over two positions in a 50:50, and despite copious recrystallisation efforts the *R*_*int*_ was higher than desirable.

**Table 4:** **Crystallographic parameters for Ti(2,4-6)(O**^**i**^**Pr)**_**2**_

**Compound reference**	**Ti(2)(O**^**i**^**Pr)**_**2**_	**Ti(4)(O**^**i**^**Pr)**_**2**_	**Ti(5)(O**^**i**^**Pr)**_**2**_	**Ti(6)(O**^**i**^**Pr)**_**2**_
Chemical formula	C_35_H_56_N_2_O_4_Ti	C_35_H_56_N_2_O_4_Ti	C_45_H_76_N_2_O_4_Ti	C_40_H_60_N_2_O_4_Ti
Formula mass	616.72	616.72	756.95	680.80
Crystal system	Triclinic	Monoclinic	Orthorhombic	Monoclinic
*a*/Å	11.2340(14)	19.3720(7)	10.9250(7)	14.0530(2)
*b*/Å	12.4310(17)	9.6520(4)	14.7200(12)	13.5820(2)
*c*/Å	13.3470(12)	20.3950(10)	27.1040(14)	20.1480(4)
*α*/°	83.037(7)	90.00	90	90
*β*/°	71.297(6)	116.015(2)	90	91.5280(10)
*γ*/°	89.540(5)	90.00	90	90
Unit cell volume/Å^3^	1751.5(4)	3427.0(3)	4358.8(5)	3844.24(11)
Temperature/K	150(2)	150(2)	150(2)	150(2)
Space group	$P\bar{1}$	*P*2_1_*/a*	*P*2_1_2_1_2_1_	*P*2_1_*/n*
*Z*	2	4	4	4
No. of reflections measured	36743	21094	52730	66001
No. of independent reflections	7965	5422	6899	6743
*R*_*int*_	0.0877	0.1729	0.1339	0.1218
Final *R*_*1*_ values (*I* > 2*σ*(*I*))	0.0564	0.0837	0.0607	0.0503
Final *wR*(*F*^2^) values (*I* > 2*σ*(*I*))	0.1265	0.1960	0.1452	0.1156
Final *R*_*1*_ values (all data)	0.1066	0.1272	0.0918	0.0830
Final *wR*(*F*^2^) values (all data)	0.1497	0.2254	0.1651	0.1348
Goodness of fit on *F*^2^	1.034	1.079	1.039	1.027

## Abbreviations

PDI: Poly dispersity index; NMR: Nuclear magnetic resonance; PLA: Polylactide; ROP: Ring opening polymerisation.

## Competing interests

The authors declare that they have no competing interests.

## Authors’ contributions

SLH carried out the work, MDJ and SLH wrote the paper. MFM and MDJ performed the crystallographic work. All authors read and approved the final manuscript.

## Supplementary Material

Additional file 1 **Crystallographic data.** Crystallographic data in CIF format for complexes CCDC Nos: 951134-951137. Click here for file
